# Molecular mechanism of ischemic postconditioning in promoting diabetic ischemic brain injury repair via the microRNA‐34a–BDNF–SIX3 signaling axis

**DOI:** 10.1002/ame2.70158

**Published:** 2026-03-09

**Authors:** Ling Zhao, Chunlan Zou, Junxian Li, Yang Yang, Yuanyuan Han, Tingyu Ke

**Affiliations:** ^1^ Department of Endocrinology The Second Affiliated Hospital of Kunming Medical University Kunming China; ^2^ Institute of Medical Biology Chinese Academy of Medical Science and Peking Union Medical College Kunming China

**Keywords:** BDNF, diabetes mellitus, IPOC, ischemic stroke, miR‐34a, SIX3

## Abstract

**Background:**

The underlying mechanisms for exacerbated brain injury and poor recovery observed in patients with diabetes and ischemic stroke (IS) remain undetermined. We explored the role of microRNA‐34a (miR‐34a) in diabetic IS (DMIS) and ischemic postconditioning (IPOC)'s neuroprotective effects in tree shrews.

**Methods:**

We established a tree shrew DMIS model and exposed it to interventions, including miR‐34a inhibition (antagomir), IPOC, and miR‐34a overexpression (agomir). Infarct size and pathology were assessed via staining. Cellular/molecular changes (astrocytes, neurons, brain‐derived neurotrophic factor [BDNF], Sine oculis homeobox 3 [SIX3], proliferation, apoptosis, axon formation) were analyzed using immunofluorescence, polymerase chain reaction (PCR), and Western blotting. In vitro, miR‐34a's targeting of BDNF/SIX3 was validated, with rescue experiments testing regulation via these factors.

**Results:**

Infarct size and neuronal damage were greater in the DMIS group than in the nondiabetic IS group. miR‐34a inhibition or IPOC reduced infarcts, alleviated injury, improved cell survival, upregulated BDNF/SIX3, enhanced proliferation/axon formation, and reduced apoptosis. miR‐34a overexpression reversed IPOC's benefits. In vitro, miR‐34a directly targeted BDNF/SIX3, suppressing their expression; exogenous BDNF/SIX3 rescued neurotoxicity and restored function.

**Conclusion:**

IPOC exerts partial neuroprotection through miR‐34a downregulation, highlighting miR‐34a as a potential therapeutic target.

## Graphical abstract

Diabetes combined with ischemic stroke (DMIS) exacerbates brain infarct size and neuronal damage compared to nondiabetic ischemic stroke (IS). This study reveals that microRNA‐34a (miR‐34a) plays a key role in DMIS pathogenesis: miR‐34a directly targets and suppresses brain‐derived neurotrophic factor (BDNF) and Sine oculis homeobox 3 (SIX3), promoting neuronal apoptosis, inhibiting cell proliferation and axon formation, and worsening neurological damage. Interventions—either miR‐34a inhibition (via antagomir) or ischemic postconditioning (IPOC)—reduce infarct area, alleviate neuronal injury, and improve astrocyte/neuronal survival in DMIS. These treatments upregulate BDNF/SIX3, enhance proliferation and axon growth, and reduce apoptosis. Conversely, miR‐34a overexpression (via agomir) reverses IPOC's protective effects, exacerbating damage. Exogenous BDNF/SIX3 supplementation rescues miR‐34a‐induced neurotoxicity in vitro. Overall, IPOC exerts neuroprotection by downregulating miR‐34a, highlighting miR‐34a as a potential therapeutic target for DMIS.

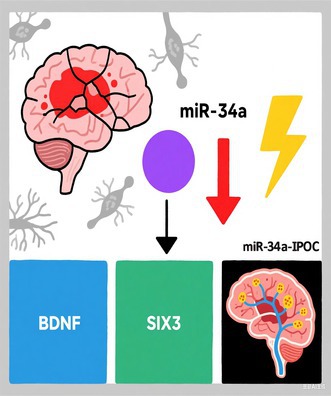

## 1 INTRODUCTION

Diabetes mellitus (DM) frequently coexists with ischemic stroke (IS), and patients with DM exhibit a worse prognosis after acute IS. A 2012–2019 study by the China Stroke Center Alliance involving 1476 centers and 838 000 patients with acute IS revealed all‐cause mortality rates of 0.8% and 0.5% in the diabetic and nondiabetic groups, respectively.^1^ Hyperglycemia exacerbates poor outcomes in patients with stroke receiving thrombolytic recanalization therapy, counteracting the benefits of early blood flow restoration.^2^ Despite progress in reducing the stroke burden over the past two decades, the rapid increase in the incidence of diabetes threatens to negate this achievement. Notably, neither intensive glycemic control during the acute stroke phase nor long‐term hyperglycemia management has demonstrated efficacy in improving outcomes or reducing stroke risk, suggesting that diabetes may worsen IS prognosis through alternative mechanisms.^3^, ^4^

Pathologically, diabetes disrupts metabolic homeostasis and impairs endogenous neurorepair mechanisms, leading to significantly poorer clinical outcomes in patients with ischemic brain injury than those observed in nondiabetic patients.^5^ Although thrombolytic therapy and neuroprotective strategies continue to advance, the complex interplay among hyperglycemia, oxidative stress, and dysregulated postischemic repair pathways has hindered the development of effective treatments for diabetic ischemic injury.^6^ Ischemic postconditioning (IPOC), a neuroprotective strategy involving intermittent blood flow restoration, is promising.^7^ However, its molecular mechanisms, particularly in the context of diabetes, remain unclear, limiting its clinical application.

MicroRNA‐34a (miR‐34a), a key regulator of cellular stress responses, is upregulated in diabetes and is strongly correlated with neuronal apoptosis and impaired synaptic plasticity.^8^ Conversely, brain‐derived neurotrophic factor (BDNF) and Sine oculis homeobox 3 (SIX3), crucial mediators of neurogenesis and axonal regeneration, are suppressed under hyperglycemic conditions.^9^, ^10^ Emerging evidence suggests that miR‐34a directly targets BDNF and SIX3, thereby forming a regulatory axis for postischemic repair.^11^, ^12^ However, whether IPOC alleviates diabetic ischemic injury by modulating this pathway remains unknown.

Our study focuses on the miR‐34a‐BDNF‐SIX3 axis within the complex multidimensional landscape of ischemic stroke mechanisms. Recent advances have highlighted multiple regulatory layers in cerebral ischemia, including noncoding RNA‐mediated blood–brain barrier protection as demonstrated by the novel peptide NP1, which confers neuroprotection through miR‐6328 upregulation and subsequent IKKβ/NF‐κB pathway inhibition.^13^ Simultaneously, membrane receptor–mediated pathways have emerged as crucial therapeutic targets, exemplified by the action of OM‐LV20 through the PAC1R/JNK/TPH1 axis to mitigate oxidative stress in astrocytes.^14^ Furthermore, epigenetic regulation of ischemic tolerance has been revealed through mechanisms such as miR‐21‐3p targeting CAMKK2‐AMPK‐Nrf2 signaling in preconditioning.^15^ Against this backdrop, our investigation of the miR‐34a‐BDNF‐SIX3 axis represents a complementary approach targeting neurorepair mechanisms in a diabetic ischemic brain.

Based on these findings, we hypothesized that IPOC mitigates diabetic ischemic brain injury by suppressing the miR‐34a‐mediated inhibition of BDNF/SIX3 signaling, thereby reactivating neurorepair pathways. In this study, we used tree shrew models that closely mimic human diabetic pathophysiology and offer distinct advantages over rodent models, combined with in vivo photochemical ischemia with IPOC intervention and in vitro high‐glucose/anoxic brain slice models, and utilized dual‐luciferase reporter assays, molecular interventions, and functional histopathological analysis to comprehensively investigate the role of the miR‐34a–BDNF–SIX3 axis in IPOC. Tree shrews were used because of their high phylogenetic proximity to primates, which is reflected in their cerebral physiology and metabolic profile. Crucially, the brain of a tree shrew exhibits microvascular density and complexity and is more analogous to the brain of humans than to the brain of rodents, making it a superior model for studying microvascular pathologies such as photochemically induced ischemic stroke.^16^ Furthermore, the immune and inflammatory responses of tree shrew brain, as well as its lipoprotein metabolism, share greater homology with humans, providing a more translatable platform for investigating the complex interplay between diabetes and cerebral ischemia.^17^ This model was designed to replicate the critical condition of acute hyperglycemia impairing stroke recovery, a scenario highly relevant to clinical settings where stroke often occurs in patients with undiagnosed or poorly controlled diabetes. The outcomes of this study may elucidate novel mechanisms of IPOC in diabetes and identify miR‐34a as a therapeutic target for enhancing poststroke recovery in metabolic disorders, potentially advancing treatment strategies for diabetic ischemic brain injury.

## 2 MATERIALS AND METHODS

### 2.1 In vivo experiments

#### 2.1.1 Establishment of the animal model

Forty‐eight tree shrews were used. The animals were acclimatized to the environment. Blood glucose levels were measured after 12‐h fasting using a Yuwell 550 blood glucose meter. Diabetes was induced by intraperitoneal injection of a 2% streptozocin (STZ) solution. The STZ solution was freshly prepared by dissolving STZ in 0.1 mol/L citrate‐sodium citrate buffer and diluting with phosphate‐buffered saline (PBS) based on the body weight of the tree shrews. A high‐fat diet consisting of 69% Tree Shrew Germplasm Resource Center basic feed, 1% cholesterol, 10% lard, 10% white sugar, and 10% fructose was provided. The production license for experimental animals was SCXK (Dian) 2024‐0002.

Cerebral ischemia was induced using the photochemical thrombosis induction (PTI) method. Under isoflurane anesthesia (induction at 3%–4% concentration with a gas flow of 0.5–0.7 L/min and maintenance at 1%–1.5% concentration), the tree shrews were fixed on a brain stereotaxic apparatus. After shaving and disinfecting the scalp, a small incision was made to expose the skull. A 1.5% Rose Bengal solution was injected via the jugular vein at a dose of 1.33 mL/kg. Ten minutes later, a specific light beam with a central wavelength of 560 nm and an intensity of 1.0 W/cm^2^ was applied for 15 min through a 0.5‐cm‐diameter hole at the marked position (1.9 mm posterior to the bregma and 4 mm right of the sagittal plane) to induce vascular endothelial damage and permanent microvascular thrombosis. A key characteristic of the PTI model was the irreversible occlusion of the microvasculature in the illuminated core region, which precluded spontaneous reperfusion.

#### 2.1.2 Randomization and grouping

After successful diabetes induction (defined as fasting blood glucose >8.0 mmol/L on two consecutive weekly measurements), eligible tree shrews were included in the study. To minimize baseline variance, animals were first stratified by sex. Within each sex stratum, they were then ranked by body weight. A computer‐generated random number sequence (created using SPSS 26.0) was applied to perform block randomization with a block size of eight. Specifically, each block of eight consecutive random numbers was assigned to the eight experimental groups in a fixed, predefined order: (1) control, (2) IS, (3) DMIS, (4) DMIS + antagomir‐NC, (5) DMIS + antagomir‐miR‐34a, (6) DMIS + IPOC, (7) DMIS + IPOC + agomir‐NC, and (8) DMIS + IPOC + agomir‐miR‐34a. This randomization method ensured an equal distribution of animals across all groups (*n* = 6 per group; *N* = 48) and balanced the groups for initial body weight, a potential confounder. The investigator performing cerebral ischemia surgery and subsequent histological/behavioral assessments was blinded to the group assignment for the antagomir/agomir and IPOC interventions.

#### 2.1.3 Interventions

In the IPOC group, a remote ischemic conditioning stimulus was applied by exposing the right common carotid artery (CCA). Notably, the CCA was not the primary site of the photochemical occlusion, which occurs distally in the cortical microvasculature. Intermittent occlusion (5 min occlusion/5 min reperfusion, repeated for three cycles) was performed using a noninvasive artery clip 30 min before the 4‐h time point after the initial cortical ischemia. This remote IPOC protocol was designed to trigger endogenous neuroprotective signaling pathways rather than to achieve direct mechanical reperfusion of the photochemically occluded territory. For molecular intervention, antagomir‐miR‐34a, agomir‐miR‐34a, and their scrambled controls (NCs; antagomir NC, agomir NC) were used. After anesthesia was induced with 3% pentobarbital (70 mg/kg, intraperitoneal injection), the tree shrews were fixed on the brain stereotaxic apparatus. A small hole was drilled at the marked position (1.9 mm posterior to the bregma, 4.0 mm right of the sagittal plane), and 5 μL of the prepared reagent was injected at a rate of 0.5 μL/min to a depth of 1.5 mm.

#### 2.1.4 Sample collection

At 24 and 72 h after cerebral ischemia, the tree shrews were deeply anesthetized with isoflurane and then sacrificed, after which their brains were collected. For triphenyltetrazolium chloride (TTC) staining, the brains were first frozen at −80°C for 5 min and then sliced into five sections with an average thickness of 2 mm. After thawing for 5 min, the slices were stained with TTC solution (prewarmed in a 37°C water bath) for 20 min in the dark and photographed immediately.

#### 2.1.5 Hematoxylin and eosin, immunohistochemical, and immunofluorescence staining

For histological analysis, brain tissue sections were processed for hematoxylin and eosin (H&E), immunohistochemical (IHC), and immunofluorescence (IF) staining: (1) H&E staining: sections were deparaffinized to water, stained with hematoxylin for 3–5 min, washed with running water for approximately 3 min to enhance the blue color, differentiated with 1% hydrochloric acid alcohol for a few seconds until the section turned red and the color became lighter, re‐blued with running water for a few seconds and then washed for 3 min, counterstained with eosin for 3–5 min, dehydrated through a series of absolute ethanol baths (5 min each in absolute ethanol I, II, III), cleared with xylene (5 min each in xylene I, II), and finally sealed with neutral gum for microscopic examination. (2) IHC staining: after deparaffinization to water, endogenous peroxidase was blocked by incubating with 3% hydrogen peroxide in a dark at room temperature for 15 min, followed by washing thrice with PBS containing Tween (PBST) for 3 min each. Antigen retrieval was performed by immersing the slides in preheated ethylenediaminetetraacetic acid (EDTA)/lemon/EDTA‐lemon antigen retrieval solution for 10 min, cooling naturally to room temperature, and washing with PBS thrice for 3 min each. Subsequently, the sections were blocked with 5% sheep serum +0.3% triton at 37°C for 30 min or at room temperature for 2–3 h. After the blocking solution was removed, the corresponding primary antibodies (BDNF at 1:100 and SIX3 at 1:100) were added and incubated overnight at 4°C or for 2 h at 37°C. After PBST washing thrice for 3 min each, the secondary antibody (horseradish peroxidase [HRP]‐labeled) was added, and the sections were incubated at room temperature for 20 min, followed by washing thrice with PBST for 3 min each. Thereafter, 3,3′‐diaminobenzidine staining solution was added for 3–5 min, and after PBST washing thrice for 3 min each, the sections were counterstained with hematoxylin for 5 min, washed with running water, differentiated, re‐blued, dehydrated with gradient ethanol (1 min each in 75%, 85%, 95% ethanol, and absolute ethanol), cleared with xylene (1 min each in xylene I, II), and sealed with neutral gum. (3) IF staining: after deparaffinization to water, the sections were washed thrice with PBST for 5 min each. They were blocked with 5% sheep serum and 0.3% triton at room temperature for 3 h. The primary antibodies (such as growth‐associated protein 43 [p‐GAP43] at 1:100, proliferating cell nuclear antigen [PCNA] at 1:200) were prepared with 5% sheep serum and incubated overnight at 4°C. After washing five times with PBST for 5 min each, the secondary antibody (fluorescently labeled) was prepared using PBST and incubated at room temperature for 3 h. After washing five times with PBST for 5 min each, the nuclei were stained with DAPI + PBS (1:3000) for 10 min or Hoechst 33342 staining solution for 10 min, after which the sections were sealed with an antifluorescence quenching mounting medium for microscopic examination and image acquisition. After IF staining, quantitative analysis was performed using ImageJ software (National Institutes of Health, USA).

### 2.2 In vitro experiments

#### 2.2.1 Cell culture

The mouse hippocampal neuronal cell line HT22 was chosen for the in vitro validation of molecular targeting and functional rescue. This choice was made because of the following reasons. First, the primary aim of the in vitro analysis was to definitively prove direct targeting and causality within the miR‐34a‐BDNF‐SIX3 axis. HT22 cells are widely used in neuroscience research and are known for their robust and reproducible response to ischemic mimics. Furthermore, they are highly amenable to efficient transfection with nucleic acids, enabling precise gain‐of‐function and loss‐of‐function experiments that are otherwise technically challenging and less efficient in primary tree shrew neurons due to lack of species‐specific genetic tools. Second, the molecular mechanism under investigation is fundamentally conserved. Notably, the seed sequence of miR‐34a is identical across mice, tree shrews, and humans. Bioinformatics analysis confirms high conservation in the putative miR‐34a binding sites within the 3′ untranslated regions (3′UTRs) of both BDNF and SIX3 genes. Furthermore, the neuroprotective roles of BDNF and the transcriptional regulatory functions of SIX3 in cell survival and development are well‐conserved biological processes in mammalian neurons. Thus, mechanistic insights derived from HT22 cells are directly relevant for the aspects of biology being studied in the tree shrew model. Third, our study employs a hierarchical “phenotype‐to‐mechanism” approach. The in vivo model of tree shrew provides essential physiological context, demonstrating the integrated neuroprotective effect of IPOC and the functional consequence of modulating the miR‐34a‐BDNF‐SIX3 axis within an intact organism that closely recapitulates key features of human diabetic stroke. The HT22 cell experiments serve a distinct, reductionist purpose: to isolate and rigorously test the specific molecular hypothesis—that miR‐34a directly targets BDNF and SIX3 mRNA to suppress their expression, and that this suppression is functionally detrimental to neuronal health. The convergence of findings from both the complex in vivo system and the controlled in vitro system significantly strengthens the validity and generalizability of the proposed signaling axis. Fourth, using a well‐characterized neuronal cell line such as HT22 to demonstrate this regulatory interaction underscores its fundamental nature as a conserved cellular pathway. This supports the translational potential of targeting this axis, suggesting relevance beyond the specific tree shrew model to other mammalian systems, including humans.

HT22 cells were cultured in high‐glucose Dulbecco's modified Eagle medium (DMEM) supplemented with 10% fetal bovine serum (FBS) and 1% penicillin–streptomycin. The cells were cultured in a humidified atmosphere containing 5% CO_2_ at 37°C. Cell resuscitation, passage, and cryopreservation were performed according to standard protocols. For cell resuscitation, the cells were rapidly thawed in a 37°C water bath and centrifuged at 1000 rpm for 3 min, and the freezing medium was discarded. Fresh complete medium was added, and the cells were transferred to a T25 flask for culture. When the cells reached 80%–90% confluence, they were passaged at a ratio of 1:3–1:4. For cryopreservation, the cells were resuspended in a cryopreservation medium comprising 90% FBS and 10% dimethyl sulfoxide, aliquoted into cryovials, and stored in liquid nitrogen.

#### 2.2.2 Determination of the optimal concentration of microRNA‐34a (miR‐34a)

Cultured brain slices were obtained from healthy shrews. After anesthesia and decapitation, the brains were quickly removed and sliced into 400‐μm‐thick sections in a 4°C oxygen‐saturated artificial cerebrospinal fluid using a vibrating microtome. Subsequently, the brain slices were placed in the upper chamber of a six‐well plate insert, with three to four slices per insert. The lower chamber was filled with 1.5 mL of high‐glucose DMEM complete medium (DMEM high‐glucose basal medium +20% horse serum +1% penicillin–streptomycin). To establish a diabetic ischemic injury model ex vivo, the slices were subjected to a combined insult of high‐glucose (25 mmol/L) medium and anoxic conditions (95% N_2_, 5% CO_2_) for 4 h at 37°C. The significant upregulation of miR‐34a and suppression of BDNF/SIX3 expression following the combined insult confirmed the validity of this model for inducing the relevant molecular pathology. The medium was then removed, and the slices were washed twice with PBS. miR‐34a mimics at different concentrations (0.1, 1, 10, and 50 μmol/L) were added to the lower chamber, and the slices were cultured overnight under the same high‐glucose and anoxic conditions.

#### 2.2.3 Dual‐luciferase reporter assay

Dual‐luciferase reporter assay was performed to verify the targeted binding relationship between miR‐34a and BDNF/SIX3. HT22 cells were seeded in 24‐well plates at a density of 2.5 × 10^4^ cells per well. When the cells reached 70%–80% confluence, Lipofectamine 3000 was used for transfection, according to the manufacturer's instructions. The cells were cotransfected with miR‐34a mimics, NC mimics, and wild‐type (WT) or mutant (Mut) BDNF/SIX3 reporter plasmids. Twenty‐four hours after transfection, the cells were lysed, and luciferase activity was measured using a dual‐luciferase reporter assay kit. The relative luciferase activity was calculated as the ratio of firefly to Renilla luciferase activity.

#### 2.2.4 Rescue experiment in vitro

Brain slices were prepared and precultured as described above. After 4‐h preculture under anoxic conditions, the medium was removed, and the slices were washed twice with PBS. Based on concentrations established in the literature for neuronal protection and axonal growth,^18^, ^19^ recombinant BDNF (50 ng/mL), recombinant SIX3 (50 ng/mL), or their combination were added to the lower chamber.

#### 2.2.5 Optimization of anoxic exposure duration

Based on preliminary experiments, the duration of high‐glucose and anoxic exposure was set at 4 h. This time frame was found to be optimal for inducing a significant and reproducible ischemic pathology in the brain slice model while maintaining sufficient tissue viability to assess the effects of molecular interventions and rescue agents in the subsequent overnight culture period. Longer exposure times resulted in excessive cell death, compromising the ability to perform functional rescue experiments.

### 2.3 Molecular biology experiments

#### 2.3.1 Reverse transcription quantitative polymerase chain reaction

Total RNA was extracted from cells or tissues using TRIzol reagent. The culture medium was removed, and the cells were washed twice with PBS. Subsequently, 500 μL of TRIzol was added, and the cells were lysed on ice for 5–10 min. For tissues, 1 mL of TRIzol was added to 50 mg of tissue, and the tissues were homogenized using a tissue grinder. After lysis, the samples were centrifuged at 12 000 rpm for 15 min at 4°C, and the upper aqueous phase containing RNA was transferred to a new tube. RNA was precipitated by adding an equal volume of isopropanol, incubated on ice for 10 min, and then centrifuged at 12 000 rpm for 10 min at 4°C. The RNA pellets were washed with 75% ethanol, air‐dried, and dissolved in RNase‐free water. The concentration and purity of RNA were measured using a microspectrophotometer.

First‐strand complementary DNA (cDNA) was synthesized using HiScript IV RT SuperMix for quantitative polymerase chain reaction (qPCR) (+gDNA wiper). The reaction system included 3 μL of 5 × gDNA wiper Mix, template RNA, and RNase‐free ddH₂O to a total volume of 15 μL, which was incubated at 42°C for 2 min. Subsequently, 5 μL of 4 × HiScript IV qRT SuperMix was added, and the reaction was performed at 37°C for 15 min and 85°C for 5 s. First‐strand cDNA was synthesized using a specific miRNA reverse transcription kit, according to the manufacturer's instructions.

qPCR was performed using the ChamQ Universal SYBR qPCR Master Mix. The reaction system included 10.0 μL of 2 × ChamQ Universal SYBR qPCR Master Mix, 0.4 μL of each primer (10 μmol/L), 2 μL of template DNA/cDNA, and ddH₂O to a total volume of 20.0 μL. The PCR cycling conditions were set according to the specific requirements of the target genes. The relative expression levels of genes were calculated using the 2^−ΔΔCt^ method, with actin or U6 as the internal reference.

#### 2.3.2 Western blotting

Tissues and cells were lysed in radioimmunoprecipitation assay lysis buffer containing protease and phosphatase inhibitors on ice for 30 min. After centrifugation at 12 000 rpm for 15 min at 4°C, the supernatant was collected, and the protein concentration was determined using a BCA protein concentration assay kit. Equal amounts of protein were mixed with 4× protein loading buffer, boiled for 5 min, and separated using sodium dodecyl sulfate‐polyacrylamide gel electrophoresis. The proteins were transferred onto nitrocellulose membranes. After blocking with 5% skimmed milk in Tris‐buffered saline with 0.1% Tween 20 detergent (TBST) for 2 h at room temperature, the membrane was incubated with primary antibodies (BDNF, SIX3, actin) overnight at 4°C. After being washed with TBST thrice for 10 min each, the membranes were incubated with HRP‐labeled secondary antibodies for 2 h at room temperature. After being washed with TBST thrice for 10 min each, the protein bands were detected using an ECL luminescence kit and imaged using a gel imaging system. The relative protein expression levels were analyzed by quantifying band intensities using the ImageJ software, with actin as the internal reference.

### 2.4 Statistical analyses

The sample size for in vivo experiments was determined a priori using G*Power 3.1 software. The primary endpoint was the neurological deficit score at 24 h postischemia. Based on our pilot data from a diabetic tree shrew ischemia model (*n* = 6 per group), the mean neurological score was 8.1 ± 1.4 (mean ± standard deviation [SD]). A systematic review of recent literature (2018–2023) on IPOC in diabetic rodent models indicated a mean reduction in the neurological scores of 2.1 ± 0.9 points. To detect a minimum clinically relevant difference (Δ) of 2.0 points with a pooled SD (σ) of approximately 1.2 (derived from our pilot and literature data), a two‐tailed independent sample *t*‐test with *α* = 0.05 and power (1 − *β*) = 0.80 required approximately six animals per group.

All data are presented as means ± SDs. Statistical analyses were performed using the GraphPad Prism software. For all multigroup comparisons, one‐way analysis of variance (ANOVA) was applied. When the ANOVA indicated a significant overall difference (*p* < 0.05), Tukey's honest significant difference post‐hoc test was used for all pairwise comparisons between groups. A *p*‐value <0.05 was considered statistically significant.

## 3 RESULTS

### 3.1 Validation of the acute diabetic hyperglycemia model

The metabolic parameters of the experimental animals are summarized in Table S1. All tree shrews subjected to STZ injection and a high‐fat diet developed sustained hyperglycemia. FBG levels in the animals in the diabetic groups (DMIS and related intervention groups) were significantly elevated at 1‐week post‐STZ and remained high at the time of stroke surgery compared to their levels at baseline and in the animals in the nondiabetic groups (control, IS) (Table S1). Body weights at baseline were comparable across all groups. At the endpoint, the mean FBG level in the pooled diabetic groups was 10.90 mmol/L, markedly higher than that in the nondiabetic control (4.28 mmol/L) and IS (4.32 mmol/L) groups. The body weight of the diabetic tree shrews at the terminal measurement was slightly lower than that of controls (134.67 g vs. 141.67 g), consistent with the catabolic state induced by acute insulin deficiency. This model effectively recapitulates the acute hyperglycemic condition known to worsen IS outcomes.

### 3.2 Inhibition of MiR‐34a significantly reduced infarct size in DMIS model tree shrews

**FIGURE 1:**
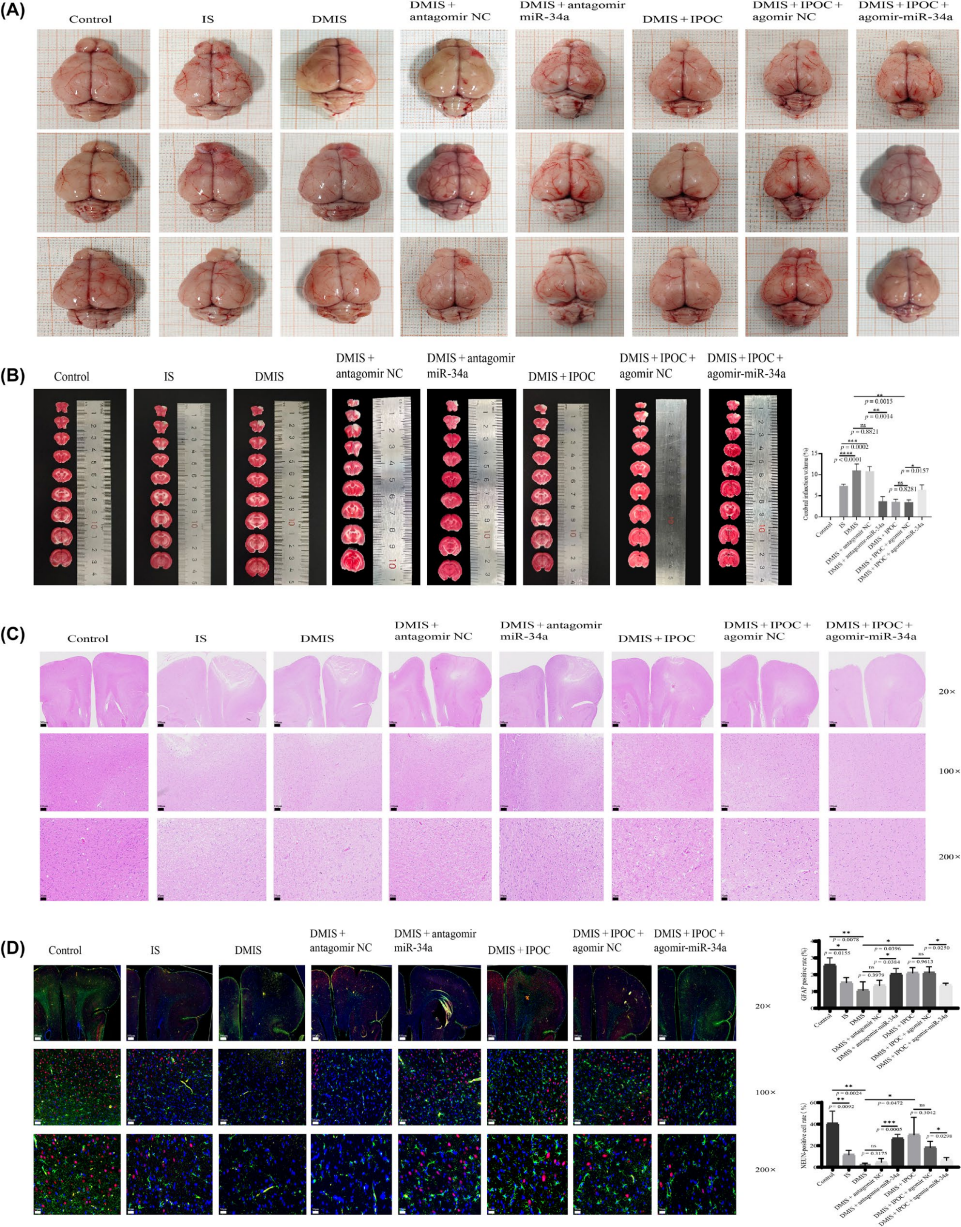
Inhibition of MiR‐34a expression reduces infarct size, improves pathological lesions, and enhances neuroprotection in diabetes mellitus–induced ischemic stroke (DMIS) model tree shrews. (A) Animal tissue sampling process in DMIS model establishment. (B) Triphenyltetrazolium chloride (TTC) staining for infarct size quantification. (C) Hematoxylin and eosin (H&E) staining for pathological changes in ischemic penumbra. (D) Immunofluorescence costaining for glial fibrillary acidic protein (GFAP) and NeuN. Data are presented as mean ± standard deviation (SD) (*n* = 6 per group). "ns" indicated "no significant difference". *p < 0.05, **p < 0.01, *** *p* < 0.001, ****p < 0.0001.

Figure S1 illustrates the process of establishing the DMIS model in tree shrews, and Figure 1A depicts the animal tissue sampling. TTC staining revealed significant differences in the infarct size among groups. Compared to the control group, both the IS and DMIS groups exhibited significantly increased infarct areas (*p* < 0.0001 and *p* = 0.0002, respectively). The DMIS + antagomir‐miR‐34a group showed a significantly lower infarct area compared to the DMIS + antagomir NC group (*p* = 0.0014). The DMIS + IPOC group also demonstrated a smaller infarct size than did the DMIS group (*p* = 0.0015), whereas the DMIS + IPOC + agomir NC group had an infarct area similar to that of the DMIS + IPOC group. Notably, the DMIS + IPOC + agomir‐miR‐34a group showed a significantly higher infarct size compared to the DMIS + IPOC + agomir NC group (*p* = 0.0157) (Figure 1B).

### 3.3 H&E staining revealed improved pathological lesions in ischemic penumbra via MiR‐34a inhibition

H&E staining was used to evaluate the pathological changes in the ischemic penumbra region of the brain tissue (Figure 1C). In the control group, the cell structures were intact with normal cell spaces, large and round cell nuclei, and no signs of edema, necrosis, or inflammatory cell infiltration. The IS group presented with swollen nerve cells, nuclear pyknosis, disordered and broken nerve fibers, tissue edema, and a small amount of inflammatory cell infiltration. The DMIS group showed more severe lesions, extensive nerve cell damage, and a strong inflammatory response. The DMIS + antagomir NC group showed evident brain tissue damage with severe neuronal injury and minimal improvement in edema and inflammatory cell infiltration. In the DMIS + antagomir‐miR‐34a group, neuronal injury was alleviated, cell morphology was relatively intact, edema was reduced, and inflammatory cell infiltration was decreased. The DMIS + IPOC group showed nearly normal neuronal morphology, a significant reduction in edema and inflammatory cell infiltration, and partial restoration of tissue structure in the ischemic penumbra, indicating the protective effect of IPOC on damaged brain tissue. The DMIS + IPOC + agomir NC group showed a similar degree of improvement as the DMIS + IPOC group, suggesting that the NC agomir NC did not have a protective effect on the brain tissue. The DMIS + IPOC + agomir‐miR‐34a group showed further reduced neuronal injury, with almost normal cell morphology, slight edema, and rare inflammatory cells; the tissue structure in the ischemic penumbra was close to normal.

### 3.4 Immunofluorescence costaining validated neuroprotective mechanism of MiR‐34a inhibition

IF costaining (Figure 1D) was used to detect the number and relative positions of astrocytes (glial fibrillary acidic protein [GFAP]) and neurons (neuronal nuclear antigen [NeuN]). Regarding GFAP, the positive rates in the IS (*p* = 0.0155) and DMIS (*p* = 0.0078) groups were significantly lower than those in the control group. The DMIS + antagomir‐miR‐34a group had a significantly higher positive rate than the DMIS + antagomir NC group (*p* = 0.0384), and the DMIS + IPOC group showed a higher positive rate compared to the DMIS group (*p* = 0.0396). The DMIS + IPOC + agomir NC group had a positive rate similar to that of the DMIS + IPOC group, whereas the DMIS + IPOC + agomir‐miR‐34a group had a significantly lower positive rate than that of the DMIS + IPOC + agomir NC group (*p* = 0.0250). Similar trends were observed for NeuN, with significant decreases in the positive rates in the IS (*p* = 0.0092) and DMIS (*p* = 0.0024) groups compared to that of the control group. The DMIS + antagomir‐miR‐34a (*p* = 0.0005) and DMIS + IPOC (*p* = 0.0472) groups had significantly higher positive rates than their respective control groups, and the DMIS + IPOC + agomir‐miR‐34a group showed a significantly lower positive rate compared to the DMIS + IPOC + agomir NC group (*p* = 0.0298). In conclusion, IF costaining revealed that inhibiting miR‐34a increased the GFAP‐ and NeuN‐positive rates in tree shrews treated with DMIS, indicating protection against neural cell damage, whereas overexpression miR‐34a reversed this effect.

### 3.5 Both MiR‐34a inhibition and IPOC enhanced BDNF and SIX3 expressions in the ischemic penumbra

**FIGURE 2:**
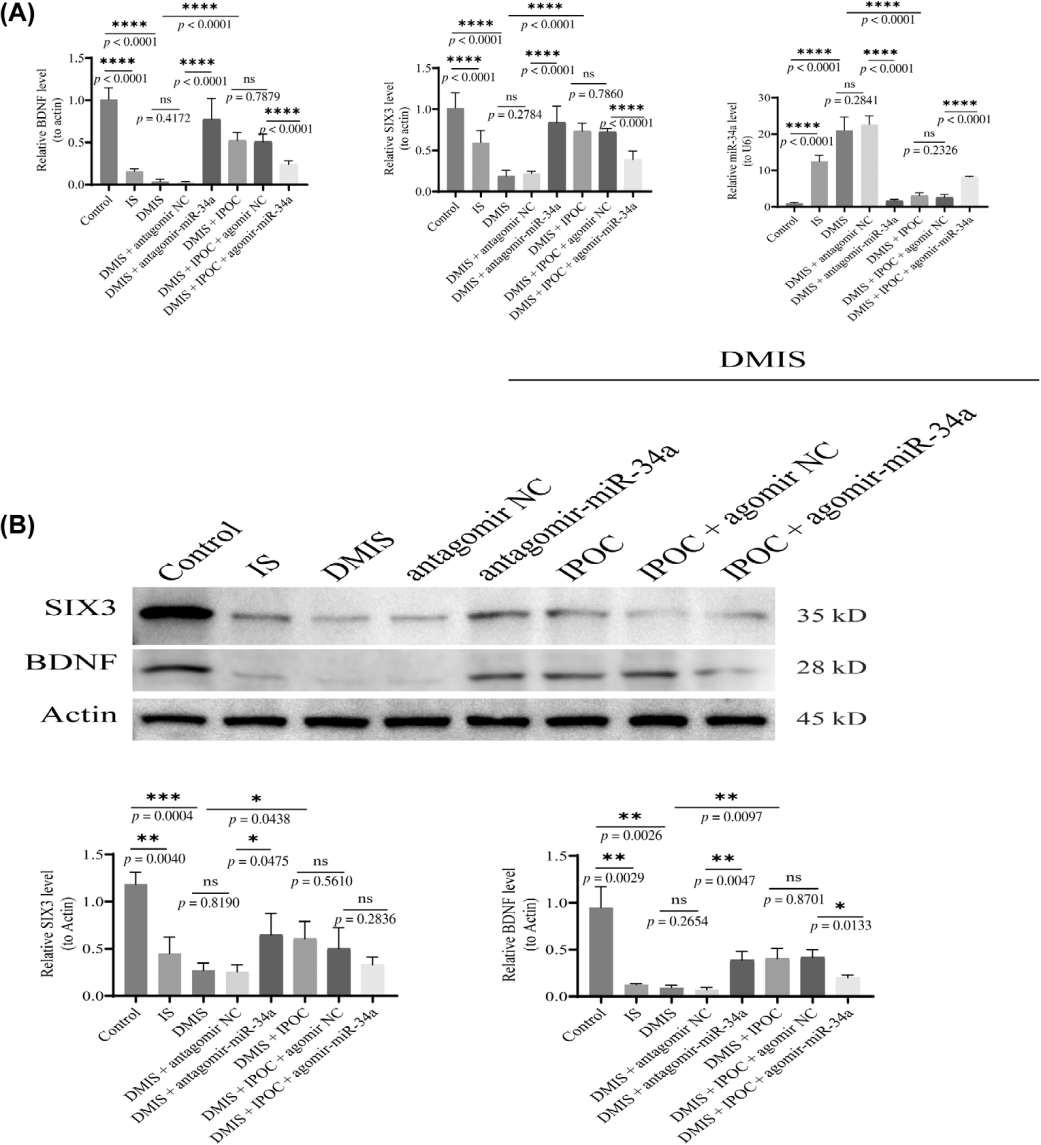
MiR‐34a inhibition and ischemic postconditioning (IPOC) upregulate brain‐derived neurotrophic factor (BDNF) and Sine oculis homeobox 3 (SIX3) expressions in the ischemic penumbra of diabetes mellitus–induced ischemic stroke (DMIS) model tree shrews. (A) Reverse transcription quantitative polymerase chain reaction (RT‐qPCR) analysis of miR‐34a, BDNF, and SIX3 messenger RNA (mRNA) expressions. (B) Representative cropped Western blot images showing BDNF and SIX3 protein expressions. Full, uncropped blots are provided in the Supplementary Information. Data are presented as mean ± standard deviation (SD) (*n* = 6 per group for in vivo experiments). "ns" indicated "no significant difference". *p < 0.05, **p < 0.01, ***p < 0.001, ****p < 0.0001.

Reverse transcription qPCR (RT‐qPCR) (Figure 2A) and Western blotting (Figure 2B) were performed to detect the expressions of miR‐34a, BDNF, and SIX3 in the ischemic penumbra region of the brain tissue. Both the IS and DMIS groups showed lower BDNF and SIX3 expressions and higher miR‐34a expression compared to the control group. There were no significant differences in the expression levels of miR‐34a, BDNF, and SIX3 between the DMIS + antagomir NC and DMIS groups. In contrast, the DMIS + antagomir‐miR‐34a and DMIS + IPOC groups showed higher BDNF and SIX3 expressions and lower miR‐34a expression compared to their respective control groups. The DMIS + IPOC + agomir‐miR‐34a group showed lower BDNF and SIX3 expressions and higher miR‐34a expression compared to the DMIS + IPOC + agomir NC group.

### 3.6 IHC validation of BDNF and SIX3 expressions

**FIGURE 3:**
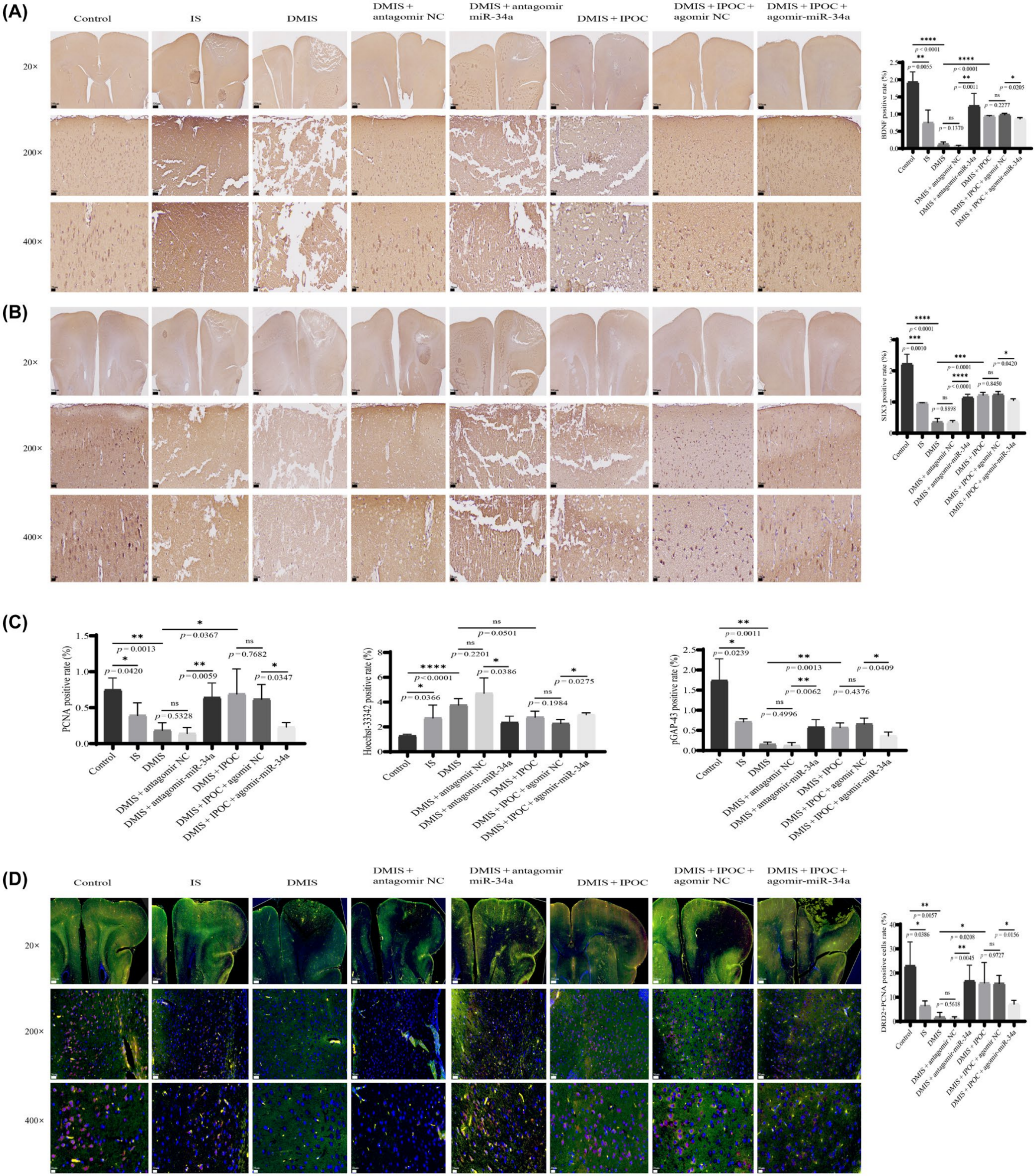
Immunohistochemistry (IHC) and immunofluorescence (IF) validation of neuroprotective mechanisms via miR‐34a inhibition and ischemic postconditioning (IPOC) in diabetes mellitus–induced ischemic stroke (DMIS) model tree shrews. (A) IHC for brain‐derived neurotrophic factor (BDNF) expression in ischemic penumbra; (B) IHC for Sine oculis homeobox 3 (SIX3) expression in ischemic penumbra. (C) IF for cell proliferation, apoptosis, and axon formation. (D) Double IF staining for DRD2/PCNA coexpression in cortical striatum. Data are presented as mean ± standard deviation (SD) (*n* = 6 per group). “ns” indicated “no significant difference”. *p < 0.05, **p < 0.01, ***p < 0.001, ****p < 0.0001.

Immunohistochemistry was used to detect the expression and distribution of BDNF (Figure 3A) and SIX3 (Figure 3B) in the ischemic penumbra of the brain tissue. The positivity rates for BDNF and SIX3 expressions in the IS and DMIS groups were significantly lower than those in the control group. The DMIS + antagomir‐miR‐34a and DMIS + IPOC groups revealed significantly higher positivity rates than did their respective control groups, whereas the DMIS + IPOC + agomir‐miR‐34a group showed a significant lower positivity rate compared to the DMIS + IPOC + agomir NC group.

### 3.7 MiR‐34a inhibition and IPOC restored cell proliferation and axon formation and inhibited apoptosis in ischemic brain tissue

IF was used to detect cell proliferation (PCNA), apoptosis (Hoechst 33342), and axon formation (pGAP‐43) in the ischemic penumbra region of the brain tissue (Figure 3C). For cell proliferation, the positive rates in the IS and DMIS groups were lower than those in the control group, whereas the DMIS + antagomir‐miR‐34a and DMIS + IPOC groups had higher positive rates than their respective control groups. The DMIS + IPOC + agomir‐miR‐34a group showed a significantly lower positivity rate compared to the DMIS + IPOC + agomir NC group. With regard to apoptosis, the positivity rates in the IS and DMIS groups were higher than those in the control group, and the DMIS + antagomir‐miR‐34a group had a lower positivity rate compared to the DMIS + antagomir NC group. The DMIS + IPOC + agomir‐miR‐34a group showed a significantly higher positivity rate compared to the DMIS + IPOC + agomir NC group. Similar trends were observed for axon formation, with the IS and DMIS groups showing lower positive rates, the DMIS + antagomir‐miR‐34a and DMIS + IPOC groups showing higher positive rates, and the DMIS + IPOC + agomir‐miR‐34a group showing a lower positive rate compared to the DMIS + IPOC + agomir NC group.

### 3.8 Dopamine receptor D2 (DRD2)/PCNA coexpression in the cortical striatum

Double IF staining was used to detect the expressions of DRD2 and PCNA in the cortical striatum (Figure 3D). The positivity rates in the IS and DMIS groups were significantly lower than those in the control group. The DMIS + antagomir‐miR‐34a and DMIS + IPOC groups had significantly higher positivity rates than their respective control groups, whereas the DMIS + IPOC + agomir‐miR‐34a group showed a significantly lower positivity rate compared to the DMIS + IPOC + agomir NC group.

### 3.9 MiR‐34a directly targeted BDNF and SIX3: Validation using the dual‐luciferase reporter assay

**FIGURE 4:**
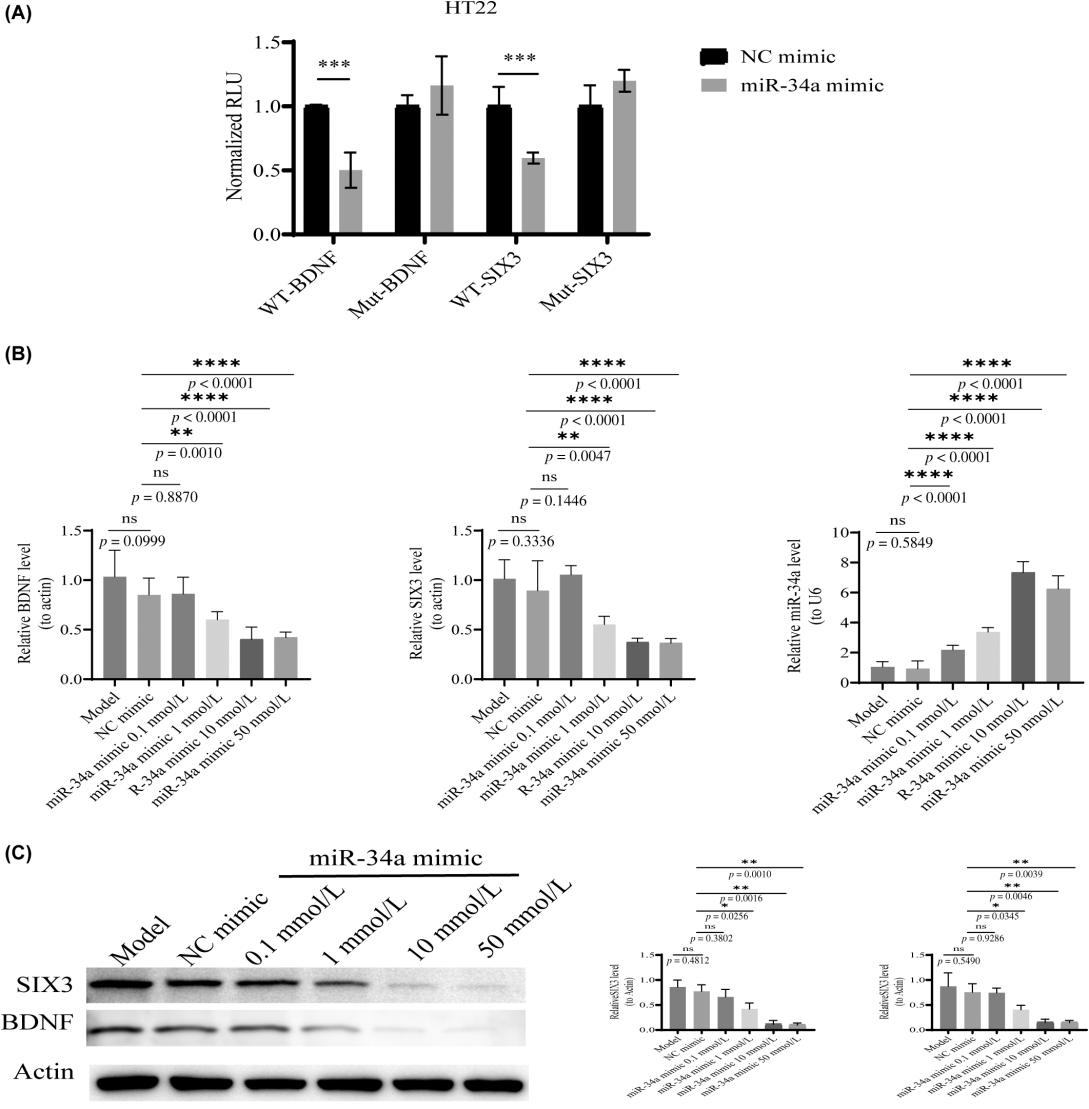
miR‐34a directly targets brain‐derived neurotrophic factor (BDNF) and Sine oculis homeobox 3 (SIX3) and regulates their expression in a dose‐dependent manner. (A) Dual‐luciferase reporter assay for miR‐34a targeting validation; (B) Reverse transcription quantitative polymerase chain reaction (RT‐qPCR) analysis of miR‐34a, BDNF, and SIX3 in striatal slices. (C) Representative cropped Western blot images showing BDNF and SIX3 protein in striatal slices. Full, uncropped blots are provided in the Supplementary Information. Data are presented as mean ± standard deviation (SD). "ns" indicated "no significant difference". *p < 0.05, **p < 0.01, ***p < 0.001, ****p < 0.0001.

The dual‐luciferase reporter assay demonstrated that miR‐34a significantly reduced the luciferase activity of WT BDNF and WT SIX3 but had no effect on the luciferase activity of Mut BDNF and Mut SIX3, confirming the targeted binding of miR‐34a to BDNF and SIX3 (Figure 4A).

### 3.10 Dose‐dependent regulation of BDNF/SIX3 by MiR‐34a in striatal slices

To further investigate the mechanism of miR‐34a‐mediated repression, we assessed the dose‐dependent effects of miR‐34a overexpression on BDNF and SIX3 expression in striatal slices. RT‐qPCR and Western blot analysis revealed that increasing concentrations of the miR‐34a mimic (1, 10, and 50 μmol/L) led to a graded upregulation of mature miR‐34a levels (Figure 4B). Crucially, this was accompanied by a parallel, dose‐dependent downregulation of both BDNF and SIX3 at the mRNA (Figure 4B) and protein levels (Figure 4C). The strong correlation and synchrony between the reduction in mRNA and protein expressions suggest that miR‐34a regulates its targets primarily by promoting mRNA instability.

### 3.11 BDNF and SIX3 mediated MiR‐34a‐driven effects on neuroplasticity‐related processes

**FIGURE 5:**
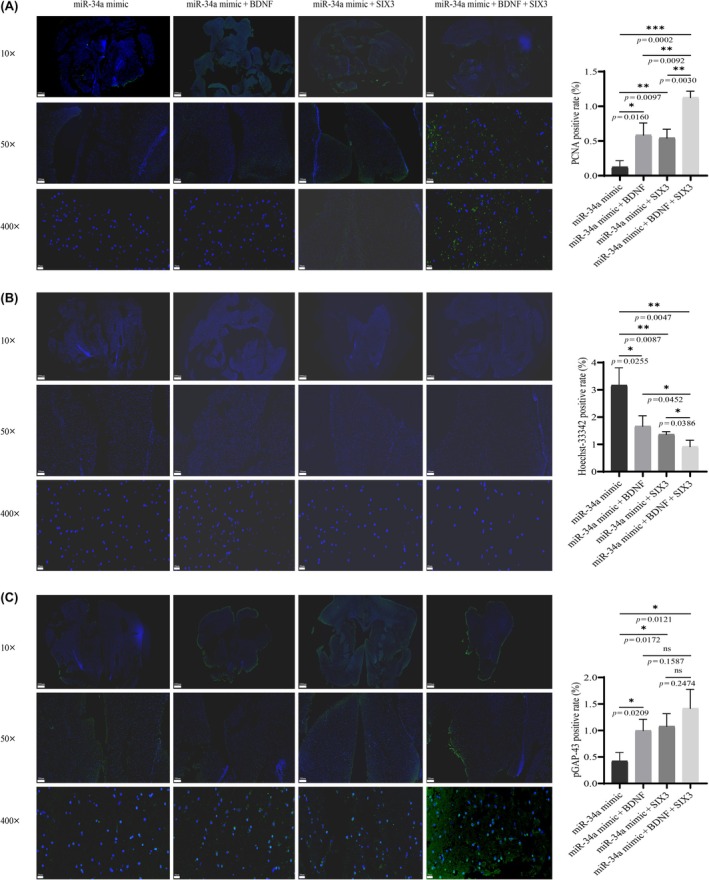
Brain‐derived neurotrophic factor (BDNF) and Sine oculis homeobox 3 (SIX3) mediate the effects of miR‐34a on neuroplasticity‐related processes in striatal tissue. (A) Immunofluorescence (IF) analysis of cell proliferation. (B) IF analysis of apoptosis. (C) IF analysis of axon formation. Data are presented as mean ± standard deviation (SD). "ns" indicated "no significant difference". *p < 0.05, **p < 0.01, ***p < 0.001.

IF was used to detect cell proliferation (PCNA), apoptosis (Hoechst 33342), and axon formation (pGAP‐43) in the striatal region (Figure 5A–C). Similar trends were observed for axon formation, with the miR‐34a mimic group showing reduced p‐GAP43 signal and the miR‐34a mimic + BDNF, miR‐34a mimic + SIX3, and miR‐34a mimic + BDNF + SIX3 groups showing higher p‐GAP43 signal compared to the miR‐34a mimic group. The IF intensity of p‐GAP43 was measured and analyzed to quantitatively support these observations. Consistent with the representative images, quantitative analysis revealed a significant decrease in p‐GAP43 intensity in the miR‐34a mimic group. This decrease was effectively rescued in the miR‐34a mimic + BDNF, miR‐34a mimic + SIX3, and miR‐34a mimic + BDNF + SIX3 groups. Additionally, there were significant differences in positivity rates among these treatment groups, indicating that BDNF and SIX3 play important roles in mediating the neuroprotective effects of miR‐34a inhibition.

## 4 DISCUSSION

This study elucidated the detrimental role of miR‐34a in exacerbating brain injury in diabetic IS and identified IPOC as a promising therapeutic strategy for counteracting miR‐34a‐mediated neurotoxicity. The use of the tree shrew model significantly strengthened the translational potential of our findings. Given the closer resemblance of the tree shrew's cerebral microvasculature to that of humans, the observed microvascular occlusion and resulting penumbral dynamics following PTI, as well as the efficacy of IPOC, are likely to be clinically more relevant than similar results obtained using standard rodent models. Similarly, the inflammatory and metabolic profile of diabetic tree shrews provides a more authentic representation of the human diabetic state. Overall, these facts increase our confidence that the identified miR‐34a–BDNF–SIX3 axis is a critical and translatable pathway in diabetic stroke pathology. Moreover, in the in vitro brain slice model, the combination of high‐glucose and anoxic conditions was sufficient to recapitulate the key molecular signature of diabetic ischemia—namely, the upregulation of miR‐34a and suppression of BDNF and SIX3. The effectiveness of our model is robustly demonstrated by these specific molecular responses and the successful execution of functional rescue experiments. However, future studies that further strengthen the characterization of this ex vivo system by incorporating direct cytotoxicity and metabolic assays, such as measuring LDH release and ATP levels, are warranted to precisely quantify the synergistic effects on cell viability.

Our findings demonstrate that hyperglycemia synergistically aggravates cerebral ischemic damage, as evidenced by the larger infarct size, severe neuronal necrosis, and heightened inflammation in the DMIS group than that observed in the IS group. Notably, our tree shrew model represents a state of acute diabetic hyperglycemia rather than a state of chronic T2DM. Although chronic models are valuable for studying long‐term complications, our acute model directly addresses the clinically critical scenario wherein stroke occurs in the setting of preexisting or acute hyperglycemia, which is a powerful independent predictor of poor outcome. The significant metabolic disturbance (severe hyperglycemia) achieved in our model is sufficient to investigate the acute molecular mechanisms underlying worsened stroke injury. These pathological changes align with the clinical observation that patients with diabetes have worse outcomes after IS, underscoring the need for targeted interventions in this high‐risk patient population.

The central discovery of our study is the dual regulatory role of miR‐34a as a molecular bridge linking hyperglycemia, neuroinflammation, and impaired recovery. Mechanistically, miR‐34a exacerbates ischemic injury by directly targeting and suppressing two key neuroprotective factors: BDNF and SIX3. The dual‐luciferase reporter assay confirmed that miR‐34a binds to the 3′UTRs of BDNF and SIX3, establishing a causal relationship between miR‐34a upregulation and their transcriptional repression. Furthermore, our dose–response experiments demonstrated a synchronous, concentration‐dependent decrease in both BDNF and SIX3 mRNA and protein upon miR‐34a overexpression. This pattern is highly characteristic of the primary mechanism of microRNA action, which involves Argonaute protein recruitment, mRNA deadenylation, and subsequent transcript degradation. Although our data cannot definitively exclude an additional, minor contribution from translational suppression, they strongly indicate that mRNA destabilization is the predominant pathway through which miR‐34a suppressed BDNF and SIX3 expression in our model.

BDNF, a critical mediator of neuronal survival and synaptic plasticity, exerts its effects primarily via the tropomyosin receptor kinase B (TrkB) pathway.^20^ TrkB binding activates a series of intracellular signaling cascades. The phosphatidylinositol 3‐kinase/Akt pathway is one of the most well‐studied downstream effectors.^21^ Akt activation leads to the phosphorylation and inactivation of proapoptotic proteins, such as Bad, while promoting the activation of antiapoptotic proteins, such as Bcl‐2,^22^ resulting in a net reduction in neuronal apoptosis. Additionally, the mitogen‐activated protein kinase/extracellular signal‐regulated kinase (MAPK/ERK) pathway is activated by BDNF/TrkB signaling.^23^ ERK translocates to the nucleus and promotes the expression of genes involved in neuronal growth, differentiation, and synaptic plasticity.^24^ In our study, miR‐34a‐mediated suppression of BDNF disrupted these critical neuroprotective pathways, leaving neurons more vulnerable to ischemic insults. SIX3, a regulator of neural development and antiapoptotic signaling, may interact with multiple cellular pathways. We hypothesize that an interaction with the Wnt/β‐catenin pathway could be a potential mechanism that needs validation through future studies.^25^ Whether SIX3 directly regulates Wnt/β‐catenin signaling through, for instance, direct protein interactions with β‐catenin or its transcriptional complexes, or operates through indirect mechanisms such as modulating the expression of pathway components, remains undetermined. Based on its known roles, SIX3 could potentially influence the stabilization and nuclear translocation of β‐catenin.^25^ In the nucleus, β‐catenin binds to T‐cell factor/lymphoid enhancer‐binding factor (TCF/LEF) transcription factors, driving the expression of genes associated with cell proliferation, survival, and neural development.^26^ Future studies employing coimmunoprecipitation and detailed pathway analysis are necessary to validate this hypothesis and clarify the precise mechanistic link between SIX3 and Wnt/β‐catenin signaling in our model. Moreover, SIX3 may interact with other transcription factors or signaling molecules involved in the regulation of mitochondrial function. By maintaining mitochondrial integrity and function, SIX3 can prevent the release of proapoptotic factors from the mitochondria, thereby reducing cell death.^27^ In diabetic IS, downregulation of SIX3 due to miR‐34a overexpression likely impairs these protective mechanisms, contributing to increased neuronal damage.

Under diabetic conditions, hyperglycemia‐induced miR‐34a overexpression disrupts this protective axis, leading to reduced astrocyte activation (GFAP), neuronal loss (NeuN), impaired proliferation (PCNA), increased apoptosis (Hoechst 33342), and axonal damage (pGAP‐43). MiR‐34a may also indirectly affect these cellular processes by modulating the expression of other genes involved in inflammation and oxidative stress. For example, miR‐34a targets genes related to the nuclear factor‐kappa B (NF‐κB) pathway, which is a central regulator of inflammatory responses.^28^ By upregulating miR‐34a, hyperglycemia may enhance NF‐κB activation, leading to the production of pro‐inflammatory cytokines, such as tumor necrosis factor‐α and interleukin‐6.^29^ These cytokines can further exacerbate neuronal injury by promoting apoptosis, inhibiting neuronal growth, and disrupting the blood–brain barrier.^30^

The functional consequences of miR‐34a dysregulation were evident in both in vivo and in vitro models. In diabetic stroke, miR‐34a inhibition restored BDNF/SIX3 expression, reduced infarct size, and improved neuronal survival by reactivating downstream neuroprotective pathways. Conversely, miR‐34a overexpression exacerbated tissue damage, highlighting its central role in the pathogenesis of diabetic stroke. Importantly, BDNF and SIX3 exerted complementary effects in vitro: exogenous BDNF enhanced neuronal proliferation and axon formation (PCNA↑, pGAP‐43↑), whereas SIX3 suppressed apoptosis (Hoechst 33342↓). Their combined rescue effect in miR‐34a‐overexpressing cells reversed neurotoxicity, underscoring their synergistic role in miR‐34a‐mediated injury. This synergy may be attributed to coordinated regulation of multiple cellular processes. For instance, BDNF‐induced activation of the ERK pathway may promote the expression of genes involved in axonal growth,^31^ whereas SIX3‐mediated stabilization of the Wnt/β‐catenin pathway may provide a permissive environment for cell proliferation and survival.^32^

IPOC has emerged as a clinically viable strategy to modulate this pathway. IPOC‐induced neuroprotection—reduced infarct size, improved neuronal morphology, and attenuated inflammation—is mechanistically associated with miR‐34a downregulation and subsequent BDNF/SIX3 reactivation. Notably, the benefits of IPOC were abolished by miR‐34a overexpression, confirming that miR‐34a suppression is a prerequisite for IPOC efficacy. This suggests that IPOC activates endogenous adaptive mechanisms, such as the miR‐34a/BDNF/SIX3 axis, to counteract hyperglycemia‐aggravated ischemic injury. One possible explanation for how IPOC downregulates miR‐34a is the activation of stress‐responsive kinases. For example, IPOC may activate protein kinase C (PKC) isoforms, which modulate miRNA expression.^33^ PKC activation can lead to the phosphorylation of transcription factors or other regulatory proteins that suppress miR‐34a transcription.^34^ Another potential mechanism involves the modulation of epigenetic markers. IPOC may induce changes in the DNA methylation or histone acetylation patterns in the miR‐34a promoter region, leading to transcriptional repression.^35^ The synergistic effects of IPOC combined with miR‐34a inhibition further support therapeutic optimization of this axis.

The role of miR‐34a in diabetic ischemic injury, beyond direct regulation of BDNF and SIX3, may be amplified through cross talk with other critical stress pathways, such as oxidative stress and dysregulated autophagy, which are hallmark features of a diabetic brain. Hyperglycemia‐induced oxidative stress is a powerful driver of miR‐34a expression via activation of transcription factors like p53 and NF‐κB. In turn, elevated miR‐34a expression can further exacerbate oxidative damage by repressing key antioxidant mediators, such as SIRT1 and Nrf2, creating a deleterious feedback loop that sensitizes neurons to ischemic insult.^36^ Concurrently, autophagy, a crucial cellular clearance process, is often impaired in diabetes. Reportedly, miR‐34a inhibits autophagic flux by targeting essential autophagy‐related genes like *ATG9A* and *BECN1*.^37^, ^38^ In our model, miR‐34a upregulation, likely triggered by the combined hyperglycemic and ischemic stress, probably contributed to the accumulation of damaged organelles and proteins, thereby compromising neuronal resilience. Therefore, the neuroprotection conferred by IPOC through miR‐34a downregulation may not only reactivate the BDNF/SIX3‐mediated repair program but also indirectly ameliorate oxidative damage and restore functional autophagy. Future studies specifically designed to dissect the temporal hierarchy and relative contribution of these interconnected pathways—neurotrophic signaling, oxidative stress, and autophagy—will be vital to fully elucidate the systemic protective mechanism of IPOC in diabetic cerebral ischemia.

Although our findings identify miR‐34a as a promising therapeutic target, some important translational questions remain. A key limitation of our study is that we did not investigate potential synergies between miR‐34a modulation and established revascularization therapies such as recombinant tissue plasminogen activator or mechanical thrombectomy. Future studies should examine whether miR‐34a inhibition can extend the therapeutic window for thrombolysis, reduce reperfusion injury, or enhance long‐term recovery when combined with these standard treatments. Such investigations will be essential for positioning miR‐34a‐targeted strategies within the comprehensive clinical management of patients with diabetic stroke. Second, systemic inhibition of miR‐34a must balance efficacy with potential off‐target effects. Given that miR‐34a is involved in various physiological processes in different tissues, nonspecific inhibition may lead to adverse effects. Third, the timing and protocol for IPOC application must be refined for patients with diabetes. The optimal time window for applying IPOC after ischemic onset may differ in patients with diabetes due to their altered metabolic state and impaired vascular function. Finally, the interplay between miR‐34a and other diabetes‐related pathways warrants further investigation.

A limitation of our current mechanistic understanding is that we did not directly measure mRNA stability or translational efficiency to conclusively dissect the precise contribution of each mechanism. The elucidation of these fine mechanistic details represents an important and logical next step for our future research. Furthermore, hyperglycemia can induce oxidative stress, which, in turn, may regulate miR‐34a expression through the activation of transcription factors. Advanced glycation end products can also interact with their receptors on neurons and glial cells, triggering a series of inflammatory and oxidative stress responses that may be intertwined with the miR‐34a/BDNF/SIX3 axis. Future studies should therefore prioritize the elucidation of the upstream regulators of miR‐34a in hyperglycemia and the downstream effectors of BDNF/SIX3 to identify additional therapeutic nodes. Understanding these complex interactions is crucial for the development of more effective and targeted therapies for diabetic IS.

Although our study provides compelling evidence for the critical role of the miR‐34a–BDNF–SIX3 axis in IPOC‐mediated neuroprotection, it is important to consider its position within the broader landscape of endogenous protective mechanisms. Reportedly, ischemic conditioning elicits a multifaceted response involving the attenuation of oxidative stress and regulation of autophagic flux.^39^ Our findings do not preclude the concurrent activation of these pathways by IPOC. In fact, potential cross talk is plausible; for instance, BDNF/TrkB signaling has been reported to activate the PI3K/Akt pathway, which can subsequently modulate oxidative stress and autophagy.^40^ Another limitation of our current study is that we did not directly measure markers of autophagy (e.g., LC3‐II/I ratio, p62) or oxidative stress (e.g., ROS levels). Therefore, we cannot definitively conclude that the miR‐34a–BDNF–SIX3 axis operates completely independently of these processes. Future studies specifically designed to inhibit this axis while measuring these parallel pathways will be essential to fully delineate the hierarchy and potential synergy between these mechanisms. Nonetheless, our data robustly demonstrate that targeted manipulation of this specific axis is sufficient to significantly alter functional outcomes, establishing its fundamental importance.

Another consideration for our in vitro rescue approach is that we used a single, literature‐based concentration of recombinant proteins. Future studies, including full dose–response curves to precisely determine the optimal and most efficacious concentrations of BDNF and SIX3 in this specific model, are warranted. Additionally, neutralizing antibodies should be employed to further confirm the specificity of the observed effects. Moreover, although our study provides comprehensive evidence of neuroprotection through multiple cellular markers, the current analysis of apoptosis and proliferation utilized well‐established single‐marker approaches (Hoechst 33342 and PCNA, respectively). Future studies incorporating dual‐labeling techniques, such as TUNEL+NeuN for specific identification of apoptotic neurons and Ki67 + DCX for precise quantification of neurogenic proliferation, are warranted as these will provide a more refined characterization of the cellular mechanisms underlying the protective effects of miR‐34a inhibition and IPOC in diabetic ischemic injury.

Although our findings demonstrate the fundamental therapeutic potential of targeting the miR‐34a‐BDNF‐SIX3 axis, several translational considerations require further investigation. Our study employed a permanent ischemia model that does not allow for exploration of post‐reperfusion therapeutic windows. Future studies using transient ischemia models should systematically evaluate the efficacy of miR‐34a inhibition, both alone and in combination with IPOC, across different treatment windows including preischemia, immediate post‐reperfusion, and delayed post‐reperfusion time points (e.g., 0–6 h). Such investigations are essential for defining the optimal therapeutic window and strengthening the clinical feasibility of this approach.

Several methodological aspects of our study warrant consideration. Although our sample size was sufficient to detect statistically significant effects in our primary endpoints, future studies with larger cohorts are required for enhanced detection of more subtle effects. Our investigation focused on acute time points to elucidate early molecular mechanisms; subsequent research should examine long‐term functional recovery to fully characterize the therapeutic potential of miR‐34a modulation. Additionally, although our experimental design specifically addressed the diabetic condition, comparison with nondiabetic IPOC effects in future work could provide further insights into diabetes‐specific pathophysiological mechanisms. The use of HT22 cells for in vitro validation, though practical for mechanistic studies, should be complemented in future research with tree shrew–derived neuronal cultures to fully address potential species differences.

## 5 CONCLUSION

In conclusion, this study identified an miR‐34a‐driven mechanism in diabetic stroke, in which hyperglycemia‐induced miR‐34a overexpression silenced BDNF/SIX3, amplifying neuronal death and impairing repair. IPOC counteracts this cascade by downregulating miR‐34a expression, offering a key therapeutic strategy for mitigating acute injury and enhancing recovery. Our work delineates a previously unreported and essential pathway for IPOC's effect, which may interact with other protective mechanisms such as oxidative stress and autophagy regulation. These insights bridge the gap between diabetic metabolic dysfunction and ischemic neurodegeneration, paving the way for precise interventions in high‐risk populations.

## AUTHOR CONTRIBUTIONS

**Ling Zhao:** Data curation; formal analysis; funding acquisition; investigation; methodology; visualization; writing – original draft. **Chunlan Zou:** Data curation; formal analysis; investigation; methodology; validation; writing – review and editing. **Junxian Li:** Data curation; investigation; methodology; visualization; writing – review and editing. **Yang Yang:** Investigation; methodology; validation; writing – review and editing. **Yuanyuan Han:** Conceptualization; funding acquisition; investigation; methodology; project administration; supervision; writing – review and editing. **Tingyu Ke:** Conceptualization; funding acquisition; investigation; methodology; project administration; resources; supervision; writing – review and editing.

## FUNDING INFORMATION

This study was supported by National Natural Science Foundation of China Project (no. 82201475), Yunnan Fundamental Research Projects (no. 202201AT070242), Yunnan Fundamental Research Kunming Medical University Joint Projects (no. 202401AY070001‐0004), Yunnan Provincial High‐Level Scientific and Technological Talent and Innovation Team Selection Special‐Young and Middle‐Aged Academic and Technical Leaders Reserve Talent Project (no. 202405AC350061), Yunnan Province Xingdian Talents Support Program (Medical and Health Talents) (no. 2024‐190408), Kunming Medical University Second Affiliated Hospital Talent Training Project (no. RCTDXS‐202308), and Science and Technology Talents and Platform Program for the Cultivation of Talents in Technological Innovation (no. 202305AD160008).

## CONFLICT OF INTEREST STATEMENT

Ling Zhao, Chunlan Zou, Junxian Li, Yang Yang, Yuanyuan Han, and Tingyu Ke declare that they have no potential conflict of interest in relation to this work.

## ETHICS STATEMENT

The use of animals in this experiment was approved by the Experimental Animal Welfare and Medical Ethics Committee of Kunming Medical University. The ethical review approval number was Kmmu20220383, and the license number was SYXK (Dian) K2023‐0009.

## CONSENT FOR PUBLICATION

The manuscript is approved by all authors for publication.

## Supporting information


Data S1.
