# Supplementary material for: Molecular mechanism of ischemic postconditioning in promoting diabetic ischemic brain injury repair via the microRNA‐34a–BDNF–SIX3 signaling axis
Source: Animal Model Exp Med. 2026 Mar 9;9(6):1126–41. doi: 10.1002/ame2.70158 (PMC13383923; doi:10.1002/ame2.70158)
Supplement: Supplementary file 1 — Data S1. [file AME2-9-1126-s001.zip › ame270158-sup-0003-DataS1.docx]

**FIGURE S1. Schematic illustration of the diabetes mellitus-induced ischemic stroke (DMIS) model establishment process in tree shrews.**
