# Supplementary material for: Molecular mechanism of ischemic postconditioning in promoting diabetic ischemic brain injury repair via the microRNA‐34a–BDNF–SIX3 signaling axis
Source: Animal Model Exp Med. 2026 Mar 9;9(6):1126–41. doi: 10.1002/ame2.70158 (PMC13383923; doi:10.1002/ame2.70158)
Supplement: Supplementary file 1 — Data S1. [file AME2-9-1126-s001.zip › ame270158-sup-0002-TableS1.docx]

**Table S1. The metabolic parameters of the experimental animals**

| Variables | Control | IS | DMIS | DMIS+antagomir NC | DMIS+antagomir-miR-34a | DMIS+IPOC | DMIS+IPOC+agomir NC | DMIS+IPOC+agomir-miR-34a |
| --- | --- | --- | --- | --- | --- | --- | --- | --- |
| Baseline Body weight (g) | 141.67±5.32 | 140.67±16.40 | 134.67±11.27 | 123.17±7.88 | 120.50±13.68 | 134.67±11.31 | 128.50±10.09 | 127.83±11.30 |
| FBG levels (Baseline) | 3.53±0.63 | 3.47±0.34 | 4.32±1.27 | 4.78±0.80 | 4.05±0.75 | 4.62±1.25 | 5.42±0.59 | 5.05±0.87 |
| FBG levels (3 days) | - | - | 10.90±4.68 | 15.00±0.89 | 12.33±3.53 | 8.10±0.94 | 12.87±3.94 | 11.97±2.77 |
| FBG levels (7 days) | - | - | 18.74±6.30 | - | 10.00±0.28 | 10.22±1.40 | 14.90±0.42 | 17.23±2.61 |
| FBG levels (14 days) | 4.28±0.33 | 4.32±0.72 | 14.90 | - | 14.75±0.07 | 17.98±1.70 | - | - |
