# Supplementary figures and images for: Molecular mechanism of ischemic postconditioning in promoting diabetic ischemic brain injury repair via the microRNA‐34a–BDNF–SIX3 signaling axis
Source: Animal Model Exp Med. 2026 Mar 9;9(6):1126–41. doi: 10.1002/ame2.70158 (PMC13383923; doi:10.1002/ame2.70158)

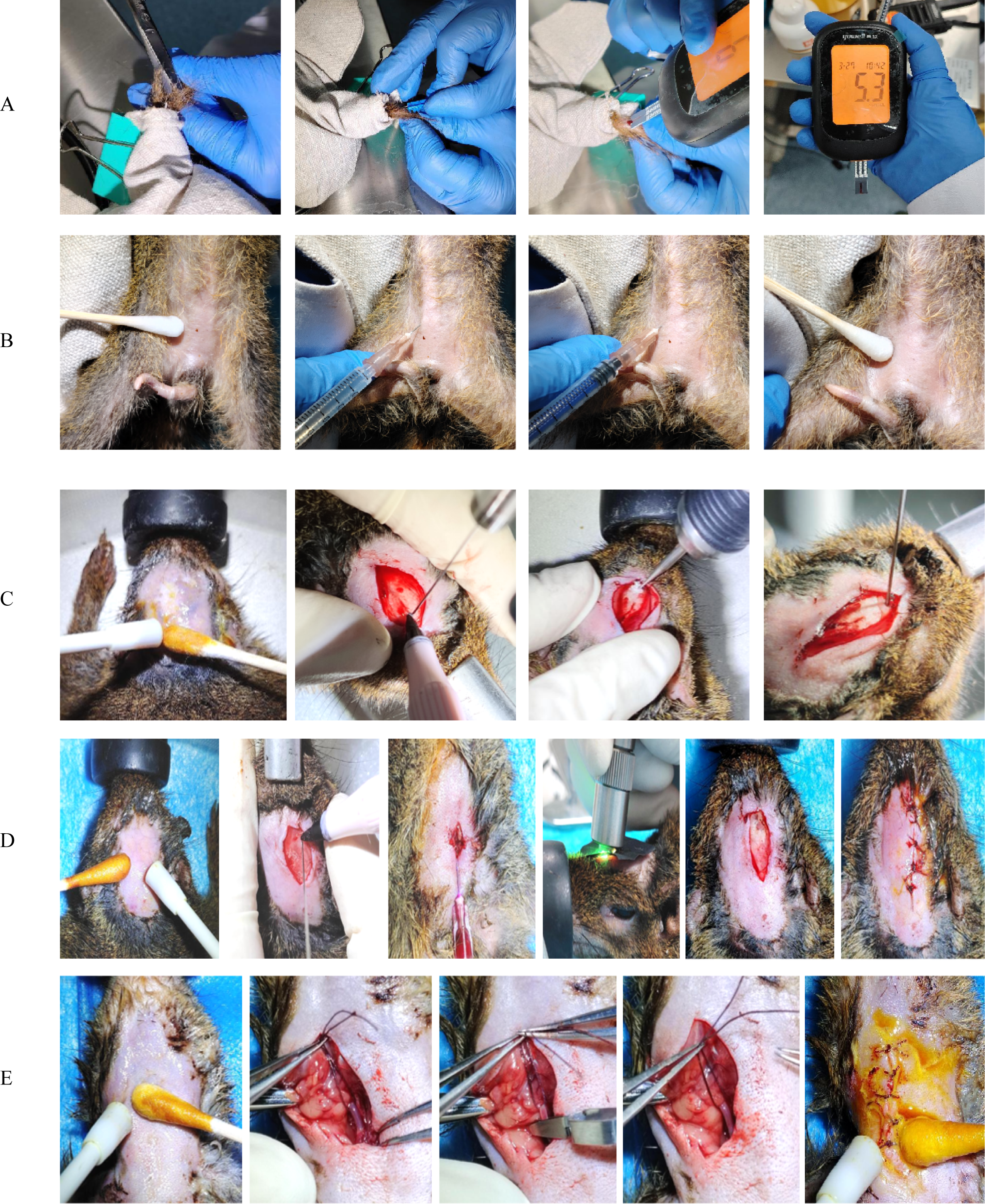

Supplement: Supplementary file 1 — Data S1. [file AME2-9-1126-s001.zip › ame270158-sup-0001-FigureS1.tif]
